# Khat (*Catha edulis* Forsk.) Dependence Potential and Pattern of Use in Saudi Arabia

**DOI:** 10.1155/2015/604526

**Published:** 2015-08-24

**Authors:** Siddig Ibrahim Abdelwahab, Rashad Mohammed Alsanosy, Bahaa-eldin E. A. Rahim, Syam Mohan, Sara Taha, Manal Mohamed Elhassan, Maged El-Setouhy

**Affiliations:** ^1^Substance Abuse Research Centre, Jazan University, Jazan 11420, Saudi Arabia; ^2^Medical Research Centre, Jazan University, Jazan 11420, Saudi Arabia

## Abstract

*Background*. *Catha edulis* Forsk. (Khat) is used for its psychoactive effects among people in Africa and the Arabian Peninsula, although its utilization is illegal in some countries such as Saudi Arabia. This study examined the pattern of Khat use and assessed the applicability of the Drug Abuse Screening Test-10 (DAST-10) to measure Khat dependence. *Methods*. A pretested questionnaire was used to gather data from 603 respondents. Variables included demographic characteristics, pattern of use, reasons for Khat chewing, and DAST-10. Stepwise-logistic regression was used to explore predictors of Khat dependence. *Results*. The majority of the respondents were married, had a secondary school level of education, were employed, were younger than 35 years old, and were living in rural areas. Many chewers gave more than one reason for using Khat. It was mainly used to increase mental capacity, physical strength, and social entertainment, as well as enhance cheerfulness and orgasms. Statistical modeling of Khat dependence suggested that the most significant predictors were residence (OR = 1.67, *P* < 0.02), frequency of Khat chewing (OR = 4.8, *P* < 0.01), age of starting Khat chewing (OR = 1.15, *P* < 0.01), and time of Khat effect (OR = 1.15, *P* < 0.04). *Conclusion*. Our study provides important information on the pattern of Khat use and its potential to cause dependence.

## 1. Introduction

*Catha edulis* Forsk. (Khat) is a flowering tree that grows wildly and is also cultivated commercially in East Africa and the Arabian Peninsula. Khat use in Southern Saudi Arabia and Yemen has become a prominent and widespread habit. The habit of chewing Khat to experience its euphoric and psychostimulant effects has prevailed for centuries among the inhabitants of the Horn of Africa and the Arabian Peninsula. Regular users of this plant get a sense of comfort, mental attentiveness, and pleasure. Postchewing effects are usually insomnia, numbness, and poor concentration. The extreme use of Khat may make substantial family, healthiness, and financial problems [1, 2]. Unfortunately, this habit has become an international issue [3, 4] as a result of the spread of Khat to western countries [5]. Cathine, cathinone, and norephedrine are the major active constituents of Khat. These chemicals are structurally associated with amphetamine and noradrenaline. Khat has a wide range of effects that may involve many organs in the human body, ranging from controversial psychic reactions to elevations in normotension, disorders of the GIT, and renal and hepatic cells toxic effects [3, 6]. A short term study conducted for over three months by Al-Mamary et al. showed that Khat has a toxic effect on the liver, which was evidenced by high serum biomarkers of liver and histopathological events [7].

In 1965, the WHO noted the abuse of Khat as a regional problem that should be controlled in Somalia, Kenya, Ethiopia, Saudi Arabia, and Yemen. Accordingly, Khat was not classified under the Single Convention on Narcotic Drugs. Khat chewing is not lawfully legalized in most of the countries, such as Yemen, Ethiopia, Somalia, Djibouti, and Kenya, where the habit is performed, although it is illegal in Saudi Arabia, Malaysia, and USA [8]. In 1980, the WHO categorized Khat as a substance of abuse that can cause mild to moderate psychological dependence. Although the WHO does not classify the dependence of Khat as a serious matter [9, 10], we noted that these conclusions were not drawn based on studies that used standard psychometric instruments. However, animal models have been widely used to investigate the dependence behavior and potential toxicological features of Khat. Research on Khat potential dependence and behavioral and mental effects in human subjects is not that widespread within the concern research entities. Numerous accessible studies have been done only in the circumstance of observation and single-case research. The literature shows that there are many studies that have stimulated the dependence behavior and toxicity in animals in response to Khat constituents [6, 11]. All these studies have agreed that developmental toxicities of Khat's addictive constituents are dose-related. The effect of cathinone on dopaminergic firing and dopamine metabolism was extensively studied in the rat brain and at the cellular level of rabbit heart [12–16]. These efforts had conclusively addressed psychostimulation and the claimed potent reinforcing properties of Khat constituents. Kassim et al. used the Severity Dependence Scale for Khat use (SDS-Khat) to assess the psychological dependence of Khat and to assess its reliability and validity in a sample of UK-resident male adult Yemeni chewers [17].

Obviously, in terms of pharmacological and toxicological impacts, four decades of intense experimental and clinical research on Khat have established a rich database. Although the scientific database on Khat is reasonably widespread and many studies have documented the potential adverse impacts of constituents of Khat on mental and psychiatric health, few community studies exist to substantiate those statements in Saudi Arabia. With exception to the animal model studies [18–21], currently, most of the studies investigating the use and dependence of Khat in Saudi Arabia are descriptive and have evaluated its prevalence [22–28]. Therefore, an immediate research effort is needed to investigate this substantial local-scale gap in knowledge in regard to the patterns of Khat use and its potential dependence among chronic Khat abusers in the Jazan region. The applicability and suitability of the Drug Abuse Screening Test-10 (DAST-10) to measure Khat dependence is based on the previous use of this psychometric instrument in evaluating abused herbs [29, 30].

## 2. Materials and Methods

### 2.1. Study Design and Sampling

This study used a community-based and cross-sectional design. The duration of the study was from December 2012 to November 2013. Participants (15 years and above) who used Khat once in the past 12 months were included in the study. Participants of either sex were included in the study. This study was approved by the Institutional Review Board of Jazan University, Jazan, Saudi Arabia. Informed written consent from the next of kin, caretakers, or guardians on behalf of the minors (15–18 years old) enrolled in this study was obtained. Some of the participants did not complete the written consent form, and those who gave verbal informed consent to willingly and voluntary participate in the study were included. During the conduction of the study, participants were informed that all the gathered data would be reserved confidentially; contribution was totally voluntary, and they had the right to leave the research at any occasion. Questionnaire was managed by medically educated personnel to guarantee the best level of data integrity and participant's confidence. Participants and students, upon agreement, were asked to tick on a box in the first page of the questionnaire indicating their willingness to take part in this study. This also helped to safely document the verbal consent. Data of the study was anonymously kept to protect participant privacy and confidentiality. Exclusion criteria included individuals who were very ill and unable to complete the study questionnaire, were younger than 15 years of age, refused to give informed consent, and were eligible participants who participated in the pretesting and test-retest of the questionnaire. As the present study is dealing with an illegal habit, authors were able to get the required approval by the respective institutional or governmental ethical committees.

### 2.2. Instrument and Variables

The DAST-10 instrument, which is written in English, was forward and backward translated by two independent bilingual translators who speak Arabic and English. Backward translation was performed to ensure that the original meaning of the questions was maintained even if the questions underwent several repetitions of the translation process. The study variables included sociodemographic variables such as age, marital status, educational level, occupation, and income. Additional important variables included information on the history of the use of Khat and information on dependence. All the wording of the DAST-10 was maintained except the name of the drug was changed to Khat. A pilot study was conducted on 36 respondents to pretest the first draft of the questionnaire. Questionnaire was modified based on the recommendations of the pretest results, and the data of the pilot study were not included in the last analysis.

### 2.3. Data Analysis

Collected data were statistically analyzed using SPSS-20 (IBM Inc., USA). Data were described statistically using means, frequency, and percentages that were appropriate. Mean differences were accordingly assessed with the suitable inferential statistical test. Cronbach's alpha (*α* > 0.7) was used to determine the internal consistency and reliability of the study instrument (DAST-10). Variables included in the logistic regression model were marital status, education level, occupation, age (categorical), residence, age of starting Khat chewing, chewing experience (years), time of chewing session, frequency of use, and postsession action of Khat (time in hours). An exploratory univariate analysis led us to use stepwise-logistic regression. The stepwise-logistic regression uses automatic method to determine the addition and removal of variables from the model. As data-driven methods, stepwise procedures are at risk of modeling noise in the data and are considered useful only for exploratory purposes [30]. A probability of *P* < 0.05 was considered statistically significant.

## 3. Results

### 3.1. Sociodemographics

A total of 603 respondents participated in this study. The response rate was 97%. Except for one female, all the participants were males. The sociodemographic uniqueness of the sample is listed in Table 1. With the exception of 6.6% Yemenis, all the respondents were of Saudi nationality. The majority of the respondents was married, had a secondary school level of education, was employed, was younger than 35 years old, and was living in rural areas (Table 1).

### 3.2. Reasons for Khat Chewing

Many chewers provided more than one rationale for using Khat (Table 2). It was mainly used to increase mental capacity, physical strength, and social entertainment, as well as enhance cheerfulness and orgasm. The majority of the respondents (85.7%) stated that Khat caused insomnia. However, respondents were divided on their opinion regarding the use of Khat to increase sexual ability. Only 2% of the respondents were using Khat for medicinal uses as shown in Table 2.

### 3.3. Chewing Patterns

The current study also investigated the chewing pattern of Khat as shown in Table 3. The mean duration of Khat use was 15.13 (SD = 9.582) years, and the mean age of starting Khat was 18.5 (SD = 3.16) years. The average effects of Khat were stated to end after 2.23 (SD = 1.4) hours. The frequency of Khat use ranged from daily, weekly, and monthly to irregularly. The results revealed that 40% of the respondents chew Khat daily as shown in Table 3.

Approximately 41% of the respondents chew Khat on a daily basis. This study included frequency of use (72 daily users and 174 nondaily users) and residence (66 rural and 278 urban). A 2 × 2 chi-square test of independence was used to determine if frequency of use was dependent on residence as shown in Table 1. Given that *α* = 0.05, the results suggested dependency, *χ* ^2^ (*df* = 1, *N* = 602) = 8.13, *P* = 0.002. Moreover, the results of the chi-square test of independence with additional variables suggested a significant association (*P* < 0.05) between frequency of use, education level, occupation, and age. Almost all the respondents (99.3%) were not addicted to other substances of abuse (Table 1).

### 3.4. Dependence on Khat and the Scoring of Modified DAST-10

Table 4 shows that less than one-third of the respondents (29.5%) were able to voluntarily quit Khat. A considerable proportion of users (66.2%) did not undergo any sort of feeling of blameworthy concerning their Khat chewing. However, approximately 61% of the participants stated that their spouse (or parents) did not agree with their Khat use. Moreover, 73.0% of the respondents neglected their family because of Khat use. Scoring of DAST-10 items was performed using the Transform function in SPSS software. The mean DAST score was 3.57 ± 0.058. The degree of problems related to drug abuse was classified according to DAST-10 into four categories, namely, no problems reported (0), low level (1-2), moderate level (3–5), and substantial level (6–10), as shown in Table 4. Nonparametric tests of homogeneity showed a significant difference between various categories of DAST-10. The respondents in the moderate level of dependence showed the highest percentage (73.6%, *χ* ^2^ = 770.46, *P* < 0.00).

### 3.5. Modeling of Dependence

The results of the binomial logistic regression analysis for potential risk factors of Khat dependence are shown in Table 5. A preliminary univariate analysis was conducted to identify potential risk factors [age, gender, education level, marital status, residence, occupation, chewing experience (years), age of starting Khat chewing, postsession action of Khat (hrs), chewing specific type of Khat, and frequency of Khat chewing], followed by binomial multivariate logistic regression analysis using stepwise method. Modeling of Khat dependence was based on DAST-10 categorization (0 for problem and 1 for moderate level), suggesting that the most significant independent predictors of Khat dependence were residence (OR = 1.67, *P* < 0.02), frequency of Khat chewing (OR = 4.8, *P* < 0.01), age of starting Khat chewing (OR = 1.15, *P* < 0.01), and time of Khat effect (OR = 1.15, *P* < 0.04). Therefore, the odds of being moderately addicted to Khat compared to a low level of dependence increased by a factor of 1.67 for those who live in rural areas compared to living in urban areas, after controlling for additional variables in the model. In terms of frequency of Khat chewing, the risk of being moderately dependent increases by 4.8 (95.0% CI for OR is 1.46 to 15.78) if the respondent is a daily chewer compared to an irregular chewer. Additional variables were insignificant in the multivariate logistic regression.

## 4. Discussion

This study was designed to investigate the pattern of Khat use and assess the applicability of the DAST-10 to examine potential dependence to Khat. A pretested questionnaire was used to gather data from 603 respondents. Research-based legalization of Khat needs further efforts using standard psychometric instruments to scientifically and firmly confirm its dependence. Khat chewing is not lawfully legalized in most of the countries, such as Yemen, Ethiopia, Somalia, Djibouti, and Kenya, where the habit is performed, although it is illegal in Saudi Arabia, Malaysia, and USA [8]. Many international studies have documented the potential adverse impacts of the addictive constituents of Khat on mental and psychiatric health, but a small number of community-based researches are available to validate those statements in KSA.

Considering the participation of only one female, the current study was dominated by male participants. The lesser number of female participants was anticipated, as Khat is not frequently used amongst women [22, 31]. The low response rate of females also mirrors the common unwillingness of Saudi women to contribute in studies of this kind [23, 32]. Additionally, Khat is principally used in villages where, culturally, people would scowl upon its chewing by females. Generally, it appears that Khat chewing is less attractive to women, although, in Somali society, using of Khat has currently become more common among women of middle class and fairly educated [33, 34].

Khat chewing is a social and daily custom in Yemen and the Jazan region in KSA [24, 25]. Therefore, the current study is consistent with this fact, as almost all the respondents were Saudi and the remaining were Yemenis (6.6%). Approximately 41% of the respondents chew Khat on a daily basis. This finding was anticipated, as Khat smuggling from Yemen to KSA is not completely controlled. The efforts done by the Government to control Khat use seem to be ineffective because trade of Khat is still widespread. The current study is the first community-based investigation in KSA that addressed the issue of Khat potential dependence. Our results suggest a significant association between frequency of use and education level, residence, occupation, and age (*P* < 0.05). Our results were not in agreement with the common beliefs that people with poorer levels of education might show stronger affinity for problem-prone behavior and are therefore expected to use drugs of abuse [35]. The possible clarification for this observation is that the majority of those individuals with lower education levels tend to be unskilled laborers, and they use Khat mainly to enable them to endure difficult physical work, whereas those individuals with such educational levels chew Khat for its euphoric effects. These findings are constant with Alem et al. [36].

The current study demonstrated that Khat is mainly used to increase mental capacity, physical strength, and social entertainment, as well as enhance cheerfulness and orgasm. The acute cognitive and emotional properties of Khat were reported by users. These effects included feelings of euphoria, improved energy and attentiveness, feelings of increased physical and mind capacity, and the belief by the chewers that Khat increases their productivity [37]. The majority of the respondents (85.7%) stated that Khat caused insomnia. There have been numerous cases of Khat chewers experiencing persistent hallucinations [3]. Khat can also affect sleep, leading to rebound effects such as late awakening, decreased productivity, and daytime sleepiness [38, 39].

The DAST-10 is a concise psychometric instrument utilized regularly to evaluate potential dependence of drugs [40]. The psychoactive effects and dependence potential of* Mitragyna speciosa*, a widely used plant in the villages in Thailand and Malaysia, were examined using DAST-10 [29]. Kassim et al. used the Severity Dependence Scale for Khat use (SDS-Khat) to assess psychological Khat dependence and to assess its validity and reliability in a sample of UK-resident male adult Yemeni chewers [17]. We used the DAST-10 for its applicable questions that facilitate explanations of the social impact of Khat on chewers. Approximately 423 (70.5%) reported that they were unable to stop using Khat, which indicates a potential dependence related with its chewing. More qualitative studies may therefore be useful to explore the psychosomatic and social effects of its use. Approximately 61% of the respondents reported the complaint of their family members about use of Khat. Earlier researchers have found that friends' and parents' use of substances is highly correlated with the use of substances among students, indicating the influence of peer pressure [25, 26]. The current study also revealed that 73% of the sample neglected their family because of Khat. Our findings suggest that Khat control and prevention campaigns and programs are required to emphasize their efforts on peers and family members to decrease the prevalence of the habit and its unfavorable consequences. As shown in Table 4, 73.6% of the sample showed moderate level (DAST-10 overall mean: 3.57 ± 0.058) of degree of problems related to drug abuse. In this case, the recommended action is to counsel chewers on the health risks associated with Khat and consider possible referral for specialized assessment [41].

Modeling of Khat dependence based on DAST-10 categorization suggested that the most significant independent predictors were residence, frequency of Khat chewing, age of starting Khat chewing, and time of Khat effect (OR = 1.15, *P* < 0.04). Rural chewers tended to develop dependence compared to people living in cities (OR = 1.67, *P* < 0.04). However, the cumulative risk of residence and frequency of use was 1.76 with a 95.0% CI of 1.18 to 2.55. The current study showed that frequency of Khat chewing is a potential predictor for failure of chewing cessation. This was evidenced by the positive regression coefficient (*β* = 0.14, *P* < 0.01). Considering the effect of frequency of Khat chewing, the risk of being moderately addicted increased by 4.8 (95.0% CI for OR was 1.46 to 15.78) if the respondent was a daily chewer compared to irregular chewer. A previous study reported that more regular use was associated with psychological Khat dependence [42]. Many experimental studies with laboratory rodents have proposed that cathinone (Khat active ingredient) possesses greater probability for abuse than amphetamine and causes continual dependence with minimal withdrawal [11]. Nonetheless, cathinone is 50% less potent than amphetamine, and this factor may also reduce the effect size observed in future laboratory studies of human Khat consumption [37].

## 5. Conclusion

Regardless of inherit structural deficiencies and average psychometric properties, Khat dependence measures based on the Drug Abuse Screening Test-10 (DAST-10) have been widely used in tobacco dependence research. The DAST-10 based measures are a preliminary step towards understanding Khat dependence among chewers. Nonetheless, additional research is recommended to study Khat dependence with the assistance of a theory-driven comprehensive scale that has superior psychometric properties. With the shortage of research related to Khat dependence, such a dependence measure will help in understanding the various underlying motives associated with the continued use of Khat, in addition to providing a capable instrument to design effective Khat chewing cessation programs. Additionally, the current study attempted to measure Khat dependence and not just addiction.

## Figures and Tables

**Table 1:** Demographic characteristics of the study group and comparison between daily and nondaily users of Khat based on demographic variables.

	Khat users						Chi-squared value, *χ* ^2^, (*P* value)
	Daily user		Nondaily user		Total		
	Number	%	Number	%	Number	%	
Gender							
Male	247	41.8	344	58.2	603	99.8	NA
Female	0	0	1	0.3	1	0.2	
Marital status							
Married	198	53.7	171	46.3	379	**62.9**	69.8 (0.00)
Single	37	18.2	166	81.8	205	34.0	
Divorcee	11	57.9	8	42.1	19	3.2	
Education level							
Illiterate	11	64.7	6	35.3	17	2.8	27.4 (0.00)
Primary	14	58.3	10	41.7	25	4.2	
Intermediate	68	55.3	55	44.7	125	20.8	
Secondary	89	31.8	191	68.2	285	**47.5**	
University	64	44.1	81	55.9	148	24.7	
Occupation							
Unemployed	18	31.6	39	68.4	58	9.7	63.5 (0.00)
Student	6	7.1	78	92.9	84	14.0	
Employee	177	51.2	169	48.8	355	**59.3**	
Worker	12	27.9	31	72.1	43	7.2	
Teacher	31	54.4	26	45.6	59	9.8	
Nationality							
Saudi	225	42.7	302	57.3	538	93.4	0.06 (0.806)
Non-Saudi	17	44.7	21	55.3	38	6.6	
Age (years)							
Less than 15	1	25.0	3	75.0	4	0.7	210.8 (0.00)
15 to 20	4	11.8	30	88.2	34	5.6	
21 to 25	11	8.7	115	91.3	128	21.2	
26 to 35	39	22.9	131	77.1	176	29.1	
36 to 42	125	70.6	52	29.4	180	29.8	
More than 42	67	82.7	14	17.3	82	13.6	
Residence							
Rural	72	52.2	66	47.8	138	22.9	8.13 (0.00)
Urban	174	38.5	278	61.5	464	**77.1**	

NA: not applicable (due to violation of chi-squared test assumption and the low number of female participants).

**Table 2:** Reason for Khat chewing and perceptions of chewers and no-chewers on why people chew Khat.

	Khat use				*χ* ^2^ (*P* value)
	Yes		No		
	Number	Percent	Number	Percent	
Khat increases the mental capacity	494	82.5	105	17.5	262.62 (0.00)
Khat increases physical strength	443	74.1	155	25.9	138.70 (0.00)
Social entertainment	562	93.4	40	6.6	452.63 (0.00)
Khat increases sexual ability	308	51.3	292	48.7	0.427 (0.514)
Khat helps to sleep	86	14.3	515	85.7	306.23 (0.00)
Khat increases cheerfulness and orgasm	395	65.6	207	34.4	58.71 (0.00)

Comparison between chewers and no-chewers was conducted using *χ* ^2^ test.

**Table 3:** Pattern of Khat chewing among the study sample.

Variables	Statistics			
	Mean	SD	Minimum	Maximum
Age of starting Khat chewing (years)	18.5	3.16	9	32
Chewing experience (years)	15.13	9.582	1	49
Postsession effect of Khat (time in hours)	2.23	1.4	1	15
Chewing specific type of Khat (*N*; %)	(9; 1.5%)			
Ethnopharmacological uses of Khat (*N*; %)	(12; 2.0%)			
Frequency of Khat chewing (*N*; %)				
More than once a day	19 (3.2%)			
Daily	228 (38.5%)			
More than once a week	214 (36.1%)			
Once a month	47 (7.9%)			
Irregularly	84 (14.2%)			

**(a):** 

Questions of DAST-10	Number (%)	
	Yes	No
Have you used drugs other than those required for medical reasons?	284 (47.1)	319 (52.9)
Do you abuse more than one drug at a time?	125 (20.8)	475 (79.2)
Are you able to stop using Khat when you want to?	177 (29.5)	423 (70.5)
Have you ever had blackouts or flashbacks as a result of Khat use?	219 (36.4)	382 (63.4)
Do you ever feel bad or guilty about Khat use?	204 (33.8)	399 (66.2)
Does your spouse (or parents) ever complain about your involvement with Khat?	368 (60.9)	236 (39.1)
Have you neglected your family because of your use of Khat?	441 (73.0)	163 (27.0)
Have you engaged in illegal activities in order to obtain Khat?	49 (8.1)	555 (91.9)
Have you ever experienced withdrawal symptoms (felt sick) when you stopped taking Khat?	25 (4.1)	578 (95.9)
Have you had medical problems as a result of your Khat use (e.g., memory loss, hepatitis, convulsions, and bleeding)?	15 (2.5)	588 (97.5)

**(b):** 

DAST-10 scoring among study sample				
Degree of problems related to drug abuse according to DAST	Suggested action	DAST standard scoring system	% of study sample	*χ* ^2^ (*P* value)
No problems reported	Encouragement and education	0-1	2.0	770.46 (0.00)
Low level	Risky behavior: feedback and advice	1-2	17.3	
Moderate level	Harmful behavior: feedback and counseling; possible referral for specialized assessment	3–5	73.6	
Substantial level	Intensive assessment and referral	6–10	7.1	
Mean ± SEM	3.57 ± 0.058		100	

**Table 5:** Modeling of Khat of dependence using stepwise logistic regression (SLR).

Variables	*β*	SE	Wald	*P* value	Odd ratio	95.0% CI for OR	
						Lower	Upper
Residence							
Urban (ref.)					1		
Rural	0.51	0.22	5.60	0.02	1.67	1.09	2.54
Frequency of Khat chewing							
Irregularly (ref.)			16.18	0.00	1		
Daily	1.57	0.61	6.66	0.01	4.80	1.46	15.78
More than once a week	1.14	0.30	14.17	0.00	3.13	1.73	5.66
Once a month	0.95	0.30	10.21	0.00	2.60	1.45	4.66
Age of starting Khat chewing	0.14	0.03	21.99	0.00	1.15	1.09	1.22
Time of Khat effect	0.14	0.07	4.05	0.04	1.15	1.00	1.32
Constant	−3.83	0.64	36.35	0.00	0.02		

Modeling of Khat dependence was based on DAST-10 categorization (0 for problem and 1 for moderate level).

Hosmer and Lemeshow goodness of fit test *χ* ^2^ = 12.326;  *P* = 0.137; −2 Log likelihood ratio = 723.377.

*B*: regression coefficient; SE: standard error of *B*.
